# Evaluation of interprovider consistency in interpretation of blood culture guidelines at an academic medical center

**DOI:** 10.1017/ash.2023.305

**Published:** 2023-09-29

**Authors:** Sherif Shoucri, Tony Li-Geng, David DiTullio, Jenny Yang, Emily Fiore, Arnold Decano, Yanina Dubrovskaya, Dana Mazo, Ioannis Zacharioudakis

## Abstract

**Background:** Blood cultures are a fundamental tool in the diagnosis of infections, but they can lead to clinical confusion and waste resources when they yield false results. To optimize blood-culture orders at our institution, we developed an evidence-based clinical guideline (Fig. 1) to be used by frontline providers on nonneutropenic hospitalized adult inpatients. We retrospectively reviewed charts of patients with positive blood cultures to evaluate whether frontline providers and infectious diseases (ID) attending physicians were able to consistently interpret the guidelines to determine whether blood cultures were drawn appropriately. **Methods:** In total, 95 nonneutropenic adults with an initial positive blood culture collected while on an inpatient unit were identified through a query of the electronic medical record from January 2021 through June 2022. Patients with polymicrobial bacteremia and bacteremia due to *Enterococcus*, *Streptococcus*, and gram-positive rods were excluded. Moreover, 4 medical resident physicians reviewed all patients and 2 ID attending physicians reviewed one-quarter of cases; all were blinded to the culture results. Blood cultures were determined to be either appropriately or inappropriately performed based on our institution’s guideline. The free-marginal multirater κ statistics with 95% CIs were calculated to evaluate interrater agreement. **Results:** Baseline patient demographics are shown in Table 1. Immune compromise without neutropenia was noted in 21 of 95 patients. Most patients were at high risk for bacteremia (72%) per our institutional guideline, most of whom were septic (67.7%). Low risk for bacteremia was found in only 12.3% of reviews. Medical resident physicians, ID attending physicians, and all reviewers combined agreed on whether blood cultures were drawn appropriately or inappropriately (84.2%, 92%, and 86.4% agreement rates, respectively). The free-marginal κ statistic was highest for ID attending physicians (0.84; 95% CI, 0.62–0.78), followed by attending physicians and resident physicians combined (0.73; 95% CI, 0.56–0.90), and resident physicians alone (0.68; 95% CI, 0.58–0.78). In the 21 patients with immune compromise, the agreement rates on blood culture appropriateness remained high among all reviewers, resident physicians, and ID attending physicians were 86.6%, 90.5%, and 95%, respectively. **Conclusions:** In our retrospective study of nonneutropenic hospitalized adult inpatients, frontline providers and ID attending physicians interpreted blood-culture guidelines consistently, largely agreeing on which patients had cultures drawn appropriately. Agreement among ID attending physicians was excellent and remained substantial among medical resident physicians. Guidelines on the appropriate use of blood cultures are vital to limiting unnecessarily collected cultures, which can lead to extended length of stay and increase cost across hospital systems. Further analyses on the clinical impact of this guideline are ongoing.

**Figure:**
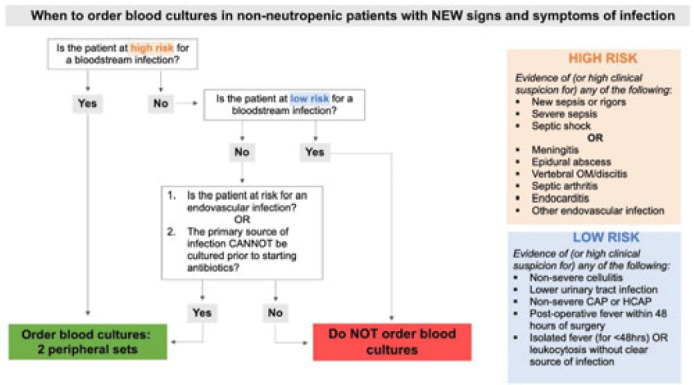

**Figure:**
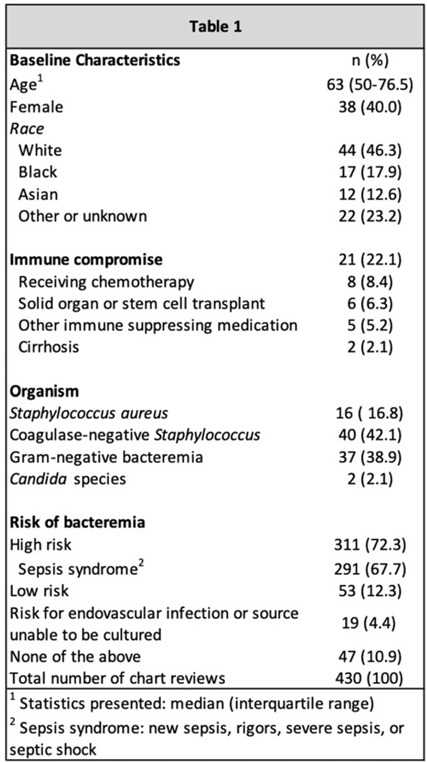

**Disclosures:** None

